# Rapid Growth of Nanocrystalline Diamond on Single Crystal Diamond for Studies on Materials under Extreme Conditions

**DOI:** 10.1038/s41598-018-19915-9

**Published:** 2018-01-23

**Authors:** Samuel L. Moore, Gopi K. Samudrala, Shane A. Catledge, Yogesh K. Vohra

**Affiliations:** Department of Physics, University of Alabama at Birmingham, Birmingham, Alabama 35294 USA

## Abstract

Early stage nucleation morphologies of spatially localized nanocrystalline diamond (NCD) micro-anvils grown on (100)-oriented single crystal diamond (SCD) anvil surfaces were analyzed and investigated for applications in high pressure studies on materials. NCD was grown on SCD using Microwave Plasma Chemical Vapor Deposition (MPCVD) for brief time intervals ranging from 1–15 minutes. Early stage film morphologies were characterized using scanning electron microscopy (SEM) and Raman spectroscopy and were compared to films grown for several hours. Rapid nucleation and growth of NCD on SCD is demonstrated without any pre-growth seeding of the substrate surface. As grown NCD diamond micro-anvils on SCD were used to generate static pressure of 0.5 Terapascal (TPa) on a tungsten sample as measured by synchrotron x-ray diffraction in a diamond anvil cell. Atomic force microscopy (AFM) analysis after decompression from ultrahigh pressures showed that the detachment of the NCD stage occurred in the bulk of the SCD and not at the interface, suggesting significant adhesive bond strength between nanocrystalline and single crystal diamond.

## Introduction

The study of materials under extreme conditions of pressures and temperatures touches a multitude of disciplines including physics, chemistry, engineering, planetary and life sciences. The implementation of diamond anvils within the confines of a diamond anvil cell in the field of high pressure research is one of the most common techniques used to generate large pressures for the study of material properties. By loading a material in an anvil cell between the culet surfaces of two diamond anvils in an opposed configuration, researchers are effectively able to ‘squeeze’ a sample, subjecting it to extreme pressures exceeding those found at the center of the earth (~360 gigapascals (GPa)). In such experiments, information on the material’s electrical, optical, and magnetic properties can be extracted through x-ray diffraction and embedded sensor probe techniques^1–3^. The ability to extend laboratory generated static pressures in the Terapascal (TPa) regime^4^ (1 TPa = 1000 GPa) using nanocrystalline diamond (NCD) micro-anvils would have a transformational impact on the fundamental understanding of materials behavior under extreme conditions.

Single crystal diamond (SCD) is the hardest known terrestrial material and widely utilized in studies on materials under extreme conditions. SCD exhibits elastic anisotropy when subjected to large strains, and manifests lower cohesion in the <111> crystallographic direction^5^, which can lead to directionally biased fracture propagation; and ultimately the catastrophic failure of the diamond anvil. Whereas nanocrystalline diamond (categorized as 5 nm < grain size <100 nm) possesses hardness comparable to that of single crystal diamond, while also demonstrating increased fracture toughness and yield strength (see study by Dubrovinskaia *et al*.^5^ and references therein). Thus, NCD’s durability makes it a highly conducive material for the next generation of diamond anvil cell devices for generation of extreme pressures whilst maintaining the structural integrity of the primary SCD anvil.

To create pressure generation tools that utilize NCD’s exceptional mechanical properties and mitigate the brittleness of SCD, methods have been employed to add a bulk nanocrystalline material “second-stage” hemispherical structure to the culet of a traditional SCD anvil^5,6^. Techniques to fabricate the NCD two-stage anvils typically involve either 1) CVD growth of nanocrystalline diamond directly onto the SCD anvil’s culet surface^6,7^; or 2) independent fabrication of a micro-ball of polycrystalline or NCD material via high temperature high pressure (HPHT) methods, prior to positioning the ball onto the culet surface of the primary SCD anvil^5,8^. In method 2 difficulties can arise in the placement of the micro-ball on the anvil culet. This process can be prone to movement and misalignment of the NCD second-stage during loading of the anvil cell, making reproducibility exceedingly difficult for experimentalists inexperienced in the methodology. Alternatively, in method 1 MPCVD is used to selectively grow nanocrystalline diamond on a localized region of the anvil culet thereby generating a second-stage that is chemically bonded to the primary SCD anvil surface. Thus far this fabrication technique has proven to be highly reproducible for the production of NCD two-stage anvils^6,7^.

Extensive research has been conducted on the polycrystalline growth of NCD on various materials such as NaCl and Silicon^9,10^; however, there is little in reported literature in regards to spatially localized NCD growth on SCD substrates. Typically in heteroepitaxial MPCVD NCD growth processes, it is required that the substrate be prepared by seeding its surface with nanocrystalline diamond crystallites that act as growth catalysts for the NCD film^11,12^. This is achieved by sonicating the substrate in a colloidal nanodiamond solution, whereby nanocrystalline particles attach to the substrate surface and in turn act as initial growth sites.

In polycrystalline films grown by seeding techniques, the final NCD film is highly dependent on the initial distribution of the attached nano-seeds to the substrate. In order to achieve thick and smooth NCD films, plasma conditions and CVD growth parameters must be altered to promote greater re-nucleation by disrupting grain growth which reduces the crystallite size^11–14^. Films grown with high re-nucleation rates that yield smaller grain sizes (<10 nm) are referred to as ultrananocrystalline, and these films’ final grain size is no longer influenced by the overall thickness of the grown film.

Although research work on spatially controlled NCD on SCD is in its early stages, our preliminary growth studies have shown remarkably consistent results^6,7^. Chemical vapor deposited NCD growth requires no seeding or preparation of the surface to promote proliferation of NCD diamond growth, and uniformity of the film is not dictated by the initial surface distribution of catalytic seeds. Thus, NCD films with consistent morphology, grain size and distribution are produced routinely, demonstrating a high level of reproducibility in fabrication.

This study establishes that rapid nucleation of NCD on SCD occurs without the use of any surface seeding to promote a particular film morphology or grain size. The structure and morphology of NCD on SCD substrates is controlled through MPCVD growth parameters and plasma chemistry, whereas seed distribution and total growth time are key factors in the final film morphology of NCD films on non-diamond substrates. The NCD micro-anvils grown in this study have been shown to generate static pressure up to 500 GPa (0.5 TPa). It is also demonstrated that the adhesive strength between the NCD film and the SCD surface is significant, with detachment of the second stage occurring in the bulk of the SCD anvil, rather than at the NCD-SCD interface.

## Methods

Growth experiments were conducted on a 1/3 carat brilliant-cut Type Ia, [100] oriented diamond anvils. Prior to CVD processing and to achieve localized NCD growth, a tungsten mask was sputter deposited onto the diamond substrate and a hole 15–20 microns in diameter was selectively etched in the center of the W-mask via maskless lithography and chemical wet-etching. The location of the etched hole influences the location of NCD nucleation, as growth only occurs in the exposed SCD region where reactive carbon growth species can form bonds with the diamond surface. NCD does not nucleate on the W-film at a significant rate, and therefore the film acts as a barrier to NCD growth on unwanted areas of the diamond anvil (see refs^3,6,7^ for further description of the tungsten masking process using maskless lithographic process). We did not observe any deterioration in NCD quality by Raman spectroscopy near the tungsten mask. The presence of the tungsten mask does not interfere with the growth of NCD on the exposed SCD surface.

A 1.2 kW magnetron tunable 2.45 GHz microwave source with a custom-built plasma chamber was used for NCD growth. Temperature was chosen as the control variable with a target value of 820 °C. Temperature measurement was performed with a two-color pyrometer that was focused through the plasma onto the diamond substrate. Plasma gas phase chemistries consisted of 9% CH_4_/H_2_ and 10% N_2_/CH_4_. Even though significant amount of nitrogen in the gas phase is utilized in NCD growth, considering 10^−4^ incorporation efficiency of nitrogen in the diamond lattice^15^, we estimate nitrogen concentration to be in 100 parts per billion in NCD. Due to the height of the diamond anvil changing slightly after each polishing, and small variations in placement in the molybdenum holding screw, chamber pressure was altered and the reactor power was adjusted in the range of 600–850 W to achieve the same temperature range for each growth experiment. Table 1 shows the total growth time and average chamber pressure for each experiment, average grain-size determined from analysis of SEM micrographs with an elliptical measurement tool in ImageJ image-processing software.

**Table 1:** Details of various growth experiments carried out for increasing time durations for a fixed.

Growth Time (min)	Pressure (Torr)	Mean Major Axis (nm)	Sigma Major Axis (nm)	Mean Minor Axis (nm)	Sigma Minor Axis (nm)
1	33	90	11	76	9
3	44	59	11	45	7
15	45	119	25	75	13
180	42	101	18	69	11
360	45	85	13	55	8

Substrate temperature of 820 °C. The growth chemistry is described in the text. Average grain sizes with standard deviation for the elliptically shaped grains are also listed.

It should be noted that although flow regulators for methane were only on for the times listed in Table 1, the cessation of methane gas flow into the reactor chamber would not immediately eliminate the presence of activated carbon growth species. However, the results clearly demonstrate the rapidity of NCD nucleation on SCD.

For analysis of growth and sample morphologies and surfaces, secondary electron SEM images were acquired with a Quanta FEG 650 Scanning Electron Microscope in high-vacuum and low vacuum modes. Accelerating voltages between 10–20 kV were used in conjunction with varying electron beam collimation sizes.

Surfaces that had experienced detachment during compression/decompression in diamond anvil cells were examined using Atomic Force Microscopy (AFM) to understand failure mechanisms. The AFM system used was a DPN5000 Nanoink system with a non-contact mode profile configuration.

Characterization of NCD films was performed via Raman spectroscopy, using a dilor XY Laser Modular Spectrometer equipped with a 50X microscope objective and a 1200 groove/mm diffraction grating. The excitation source used was a 532 nm, 300 nW frequency-doubled YAG laser that was attenuated through a series of neutral density filters (OD 1–5). A liquid nitrogen cooled PI-Acton Spec 10 detector and Winspec32 software were used to record the spectra.

Two stage anvils that were grown for 3 hours were implemented in high pressure experiments in which 1-micron thick tungsten was deposited onto the anvil surface via sputter deposition. High pressure angle-dispersive x-ray diffraction studies were carried out at the Advanced Photon Source, Argonne National Laboratory beam-line 16-ID-B. The monochromatic X-rays utilized in the diffraction experiments had a wavelength λ = 0.4066 Å. The image plate data were integrated using DIOPTAS.

The tungsten equation of state for pressure calibration was obtained from the shock data of Hixson *et al*.^16^ by fitting the third order Birch-Murnaghan equation^17^ to the pressure-volume (P-V) isotherm to 300 GPa at 293 K. The parameters obtained from shock data are bulk-modulus (B_0_) = 325.67 GPa and the pressure derivative of bulk-modulus (B_0_^′^ = 3.67). Recently, there has been an additional equation of state measurement of tungsten to static pressure of 200 GPa using gold and platinum pressure standards^18^ and parameters obtained in this study are bulk-modulus (B_0_) = 307 GPa and the pressure derivative of bulk-modulus (B_0_^′^ = 4.53). We have employed both equations of state to estimate the uncertainty in highest pressure reached in our NCD micro-anvil experiments.

All the data is available on request from the authors.

## Results

### Structure and Geometrical Growth Limit

**Figure 1:**
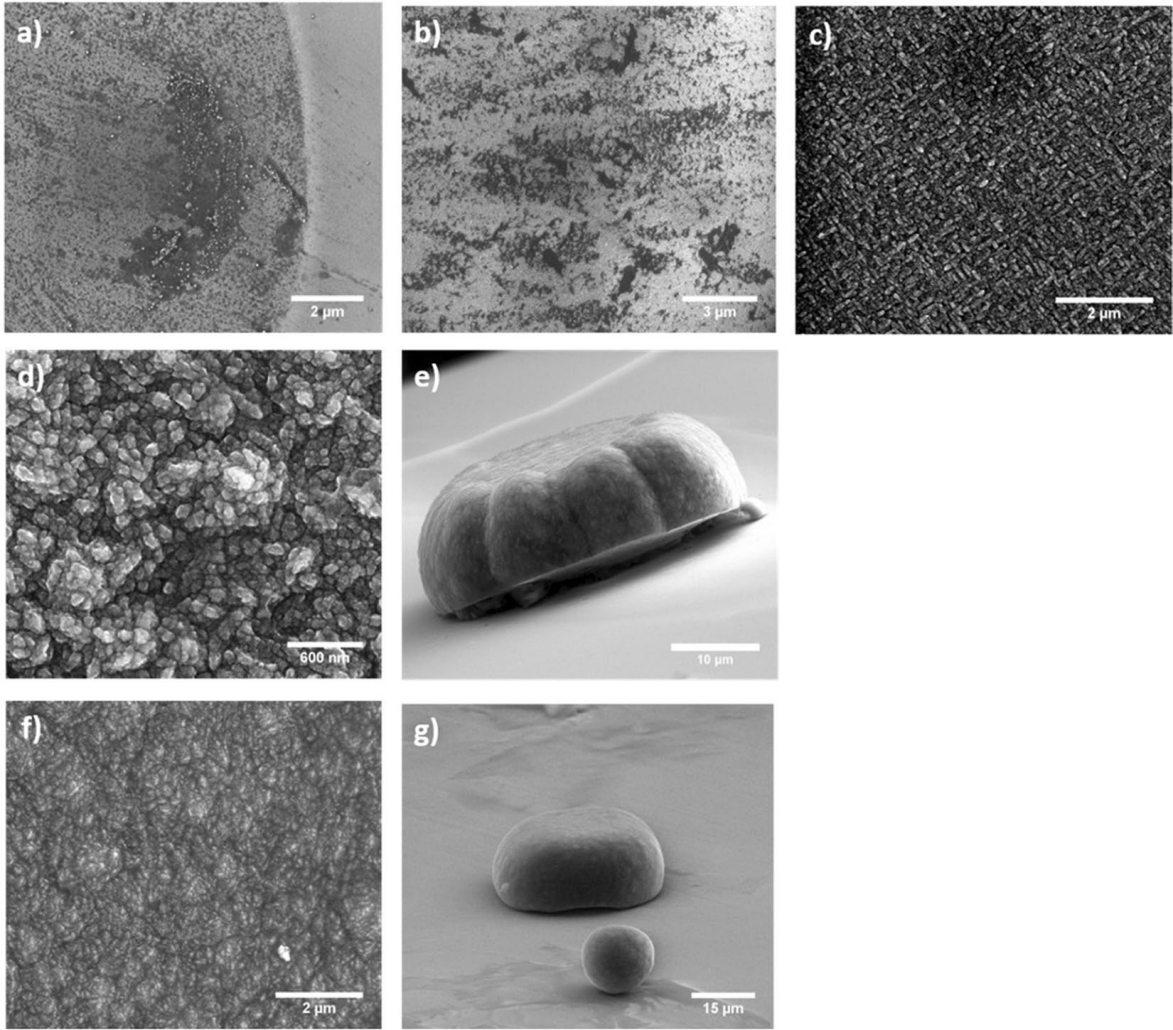
SEM images of the various growth experiments shown at different magnifications to give a broad perspective of nuclei density and coverage of the SCD surface. Growth experiments are depicted as follows: (**a**) 1 minute (**b**) 3 minutes, dark regions are SCD areas without NCD growth. (**c**) 15 minutes showing uniform coverage (**d**) 3 hours (**e**) 3 hour side view showing growth only in the region defined by tungsten mask (**f**) 6 hours (**g**) 6 hour side view showing a satellite spherical NCD growth in addition to central second stage defined by tungsten mask.

SEM images show that nucleation of NCD on the SCD surface occurs rapidly (see Fig. 1.). After only one minute of growth, there are substantial nucleation sites on the surface of the SCD anvil. After three minutes, there are only small areas of SCD without NCD coverage, and by 15 minutes there is complete and uniform coverage over the entire growth region. At three hours of growth, side-view SEM images suggest an approximate growth rate of 5 µm/hour. Morphology and structure of the second-stage does not appear to change significantly between 3 and 6 hours of growth, with both 3 and 6 hour runs exhibiting essentially the same diameter and height of the second stage. Therefore, the growth rate of 5 µm/hour observed in the 0–3 hour growth period is not constant over time. It is not currently understood what is causing this phenomenon, and suggests the possible existence of geometrical limit in the growth of the second stage. The NCD growth structure appears to favor a spherical like geometry, with second-stage growths forming a roughly hemispherical protrusion on the anvil culet surface. This geometry has been observed consistently over every two-stage growth experiment that we have performed, where second-stage geometries have invariably exhibited a hemispherical growth of approximately 30–40 µm in diameter, and a height of 15–20 µm relative to the SCD surface. The lateral dimensions of the second stage are likely dictated by the initial area of the hole in the tungsten mask. Figure 1g displays further evidence of the preferential spherical geometry of the second-stage. Pictured is an unintentional satellite growth in the image foreground that has nucleated away from anticipated growth region. Satellite growths occur due to the delamination of a small portion of the tungsten mask, and have formed into a near perfect sphere.

### NCD Characterization

**Figure 2:**
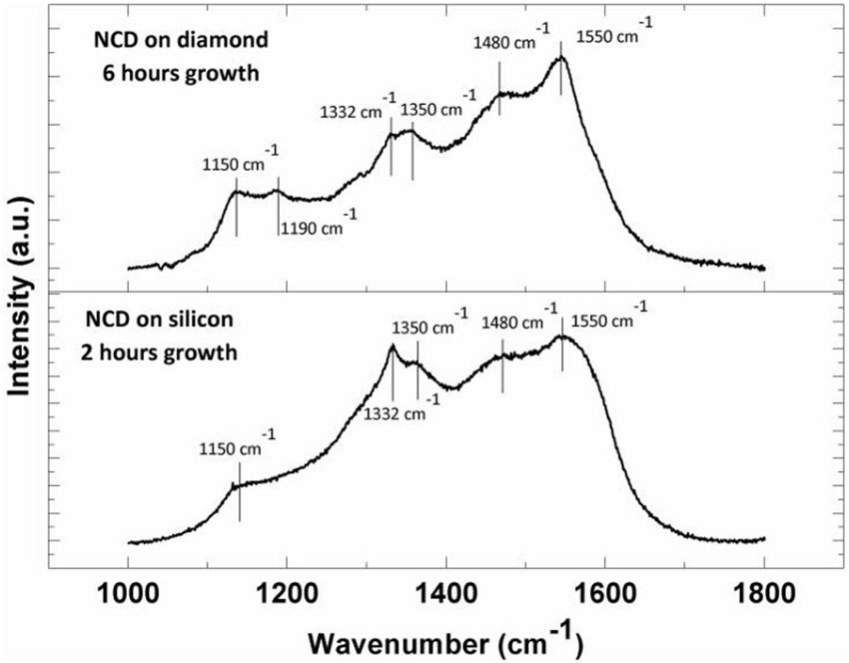
The background subtracted Raman spectra of NCD film grown for six hours on single crystal diamond and for two hours on silicon substrate. Peaks at 1150 cm^−1^, 1190 cm^−1^, 1350 cm^−1^, 1480 cm^−1^ and 1550 cm^−1^ are attributed to sp^2^ bonded carbon and transpolyacetylene, and are characteristic of NCD films. A very weak 1332 cm^−1^ Raman peak corresponding to sp^3^ bonded carbon is also observed for NCD films in both cases.

In addition to SEM microscopy, Raman spectroscopy was used for characterization of the nanocrystalline films. Due to the lack of NCD material in the shorter growth experiments, Raman spectra were dominated by sp^3^ bonded carbon peaks at 1332 cm^−1^ associated with single crystal diamond. The growth experiments of 3 hours and beyond had sufficient NCD material to show characteristic nanodiamond spectra with peaks centered at 1350 cm^−1^ and 1550 cm^−1^ attributed to amorphous carbon D and G signals, respectively; and peaks at 1150 cm^−1^, 1190 cm^−1^ and 1480 cm^−1^ attributed to sp^2^ bonded transpolyacetylene (TPA)^19^. These five peaks are a commonly observed characteristic of NCD and can be seen in Fig. 2 which displays a Raman spectrum for the 6-hour growth experiment. The Raman peak from the SCD substrate is not observed in Fig. 2 due to absorption of the incident laser beam in the 15-micron thick NCD micro-anvil. For comparison purposes in Fig. 2, we also show a Raman spectrum for NCD grown for 2 hours on silicon substrate using similar growth chemistry and plasma conditions. It is clear from Fig. 2 that Raman spectra of NCD grown on SCD and NCD growth on silicon substrate are very similar. A very weak 1332 cm^−1^ Raman peak corresponding to sp^3^ bonded carbon is also observed for NCD films in both cases. It is difficult to quantitatively measure the sp^3^-bonded carbon or diamond content in the NCD films by Raman spectroscopy. Our previous x-ray photoelectron spectroscopy studies^6^ on NCD films on SCD substrates have revealed about 72% sp^3^-bonded diamond content and 28% sp^2^-bonded graphitic content in as grown samples.

### High Pressure Results

**Figure 3:**
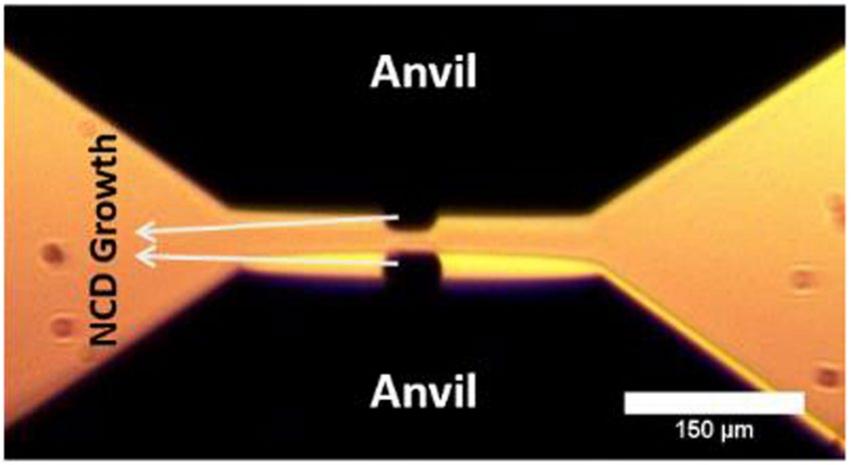
NCD micro-anvils grown on single crystal diamond anvils and mounted in an opposed anvil configuration before pressurization in a diamond anvil cell. The entire diamond anvil including the NCD micro-anvil has been deposited with a one micron thick layer of tungsten metal for pressure measurement by x-ray diffraction.

The two NCD micro-anvils grown on SCD anvils were coated with a 1 micron thick tungsten sample by sputter deposition and mounted in a diamond anvil cell in an opposed-anvil configuration as shown in Fig. 3. A soft 10-micron thick terbium foil was placed between the two anvils to provide initial spacing before applying pressure on the anvils using a gas-membrane assembly at beam-line 16-ID-B, HPCAT, Argonne National Lab. The angle dispersive x-ray diffraction to the highest pressure were recorded using x-ray wavelength of λ = 0.4066 Å.

The x-ray diffraction pattern were dominated by the sputter-deposited tungsten sample in our experiments. Our measured lattice parameter of tungsten at the highest pressure is *a = *2.6659 ± 0015 Å. The observed and calculated d-spacing values for tungsten at the highest pressure are shown in Table 2. Using ambient pressure lattice parameter of tungsten of 3.164 Å, the measured volume compression is V/V_0_ = 0.598. The following third-order Birch-Murnaghan equation of state^16^

1 $$P=\frac{3}{2}{B}_{0}\,[{(\frac{{V}_{0}}{V})}^{\frac{7}{3}}-{(\frac{{V}_{0}}{V})}^{\frac{5}{3}}]\{1+\frac{3}{4}({B}_{0}^{\text{'}}-4)[{(\frac{{V}_{0}}{V})}^{\frac{2}{3}}-1]\}$$

Using bulk-modulus (B_0_) = 325.67 GPa and the pressure derivative of bulk-modulus (B_0_′ = 3.67) gives a pressure of 423 GPa. Using (B_0_) = 307 GPa and the pressure derivative of bulk-modulus (B_0_′ = 4.53) gives a pressure of 516 GPa. Therefore, the highest pressure reached in our experiments is in the range of 423–516 GPa range considering the uncertainty in the equation of state for tungsten.

**Table 2:** The (hkl) Miller indices and observed and calculated interplanar spacing’s (d_hkl_) for x-ray diffraction pattern for body-centered (bcc) tungsten at the highest pressure.

hkl	d_hkl_ (Observed) Å	d_hkl_ (Calculated) Å
110	1.8932	1.8851
200	1.3298	1.3329
211	1.0881	1.0883

The observed lattice parameter of tungsten at the highest pressure is a = 2.6659 ± 0015 Å. The calculated pressure is in the 423–516 GPa range as described in the text.

**Figure 4:**
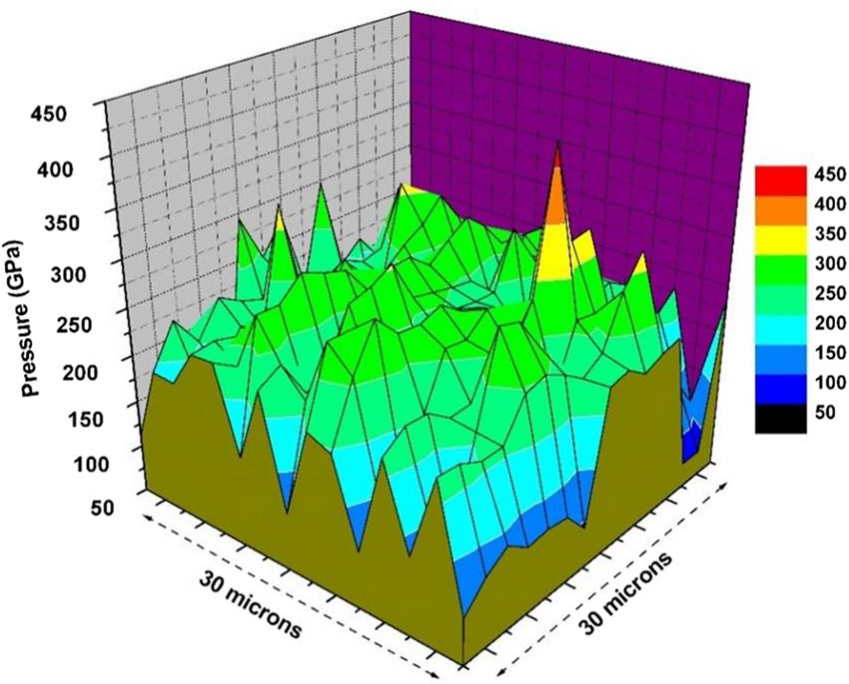
2-D pressure distribution obtained by micro-collimated x-ray diffraction at a synchrotron source. The pressures are obtained from the measured volume of tungsten and using shock equation of state^15^.

A detailed pressure distribution map on the NCD stage was obtained during an intermediate pressure by scanning the 30 micron X 30 micron area using a micro-collimated x-ray beam with 2 micron step in both directions with a total of 225 data points. The nominal size of x-ray beam during pressure distribution measurement was 1 micron (vertical) x 2 micron (horizontal). Since the tungsten pressure marker is present on the entire face of diamond anvil including the NCD micro-anvil, the pressure was calculated from the measured volume of tungsten at each location and using the known equation of state of tungsten as discussed earlier. The resulting 2-D pressure distribution map is shown in Fig. 4 at a maximum pressure of 450 GPa. It is interesting to note that the highest pressure region is largely concentrated on the NCD micro-anvil and shows significant drop in pressure at the edge of the 30 micron region of NCD growth (Fig. 4). This implies that that NCD micro-anvil has unusually high shear strength and it can support a large pressure gradient in the diamond anvil cell and hence allows us to reach very high static pressures.

### AFM Characterization of the SCD Surface after NCD Detachment

**Figure 5:**
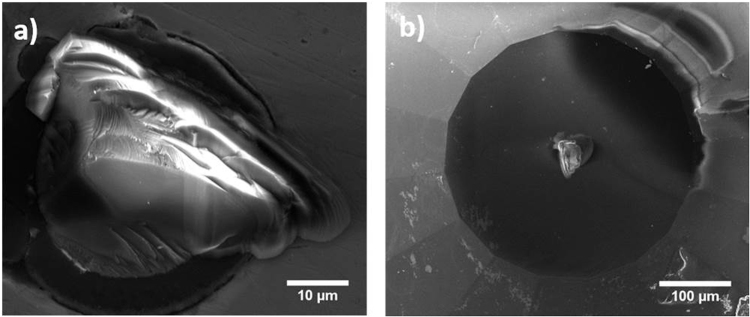
SEM images of (**a**) a detached diamond fragment from a two-stage anvil grown for three hours; (**b**) the anvil culet post detachment of the NCD growth.

**Figure 6:**
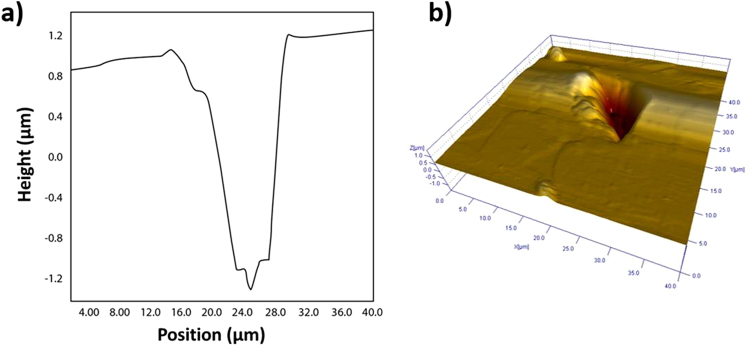
(**a**) AFM line profile scan over the trench in the diamond culet (pictured in Fig. 3b), showing a depth of approximately 2.4 µm. (**b**) AFM 40 × 40 µm area scan depicting the size of the SCD fragment that detached from the bulk of the SCD anvil.

The NCD anvils were examined after decompression to examine the detachment of second stage from the SCD anvil during release of pressure. Surprisingly, decompressed anvils that were examined under SEM appeared to experience detachment in the bulk of the SCD anvil, and not at the NCD-SCD interface as expected. It is difficult to confirm the location of detachment through SEM alone, and therefore a more exhaustive approach was taken in this study to confirm failure in the SCD bulk by examining a detached NCD two-stage anvil (grown for 3 hours under conditions listed in experimental methods section). The detached anvil and the steel gasket used during loading of an anvil cell were examined with AFM and Raman spectroscopy in conjunction with SEM. Scanning electron microscopy images in Fig. 5 show the detached diamond fragment embedded into a steel gasket (Fig. 5a.) and the culet surface of the anvil post detachment (Fig. 5b.). AFM scans (Fig. 6) indicate that upon detachment of the NCD growth, failure occurred in the bulk of the SCD anvil and not at the NCD-SCD interface. This is clearly apparent from the AFM line profile and area scans across the detachment region of the SCD culet that shows a distinctive trench with an approximate depth of 2.4 µm into the bulk of the SCD anvil.

**Figure 7:**
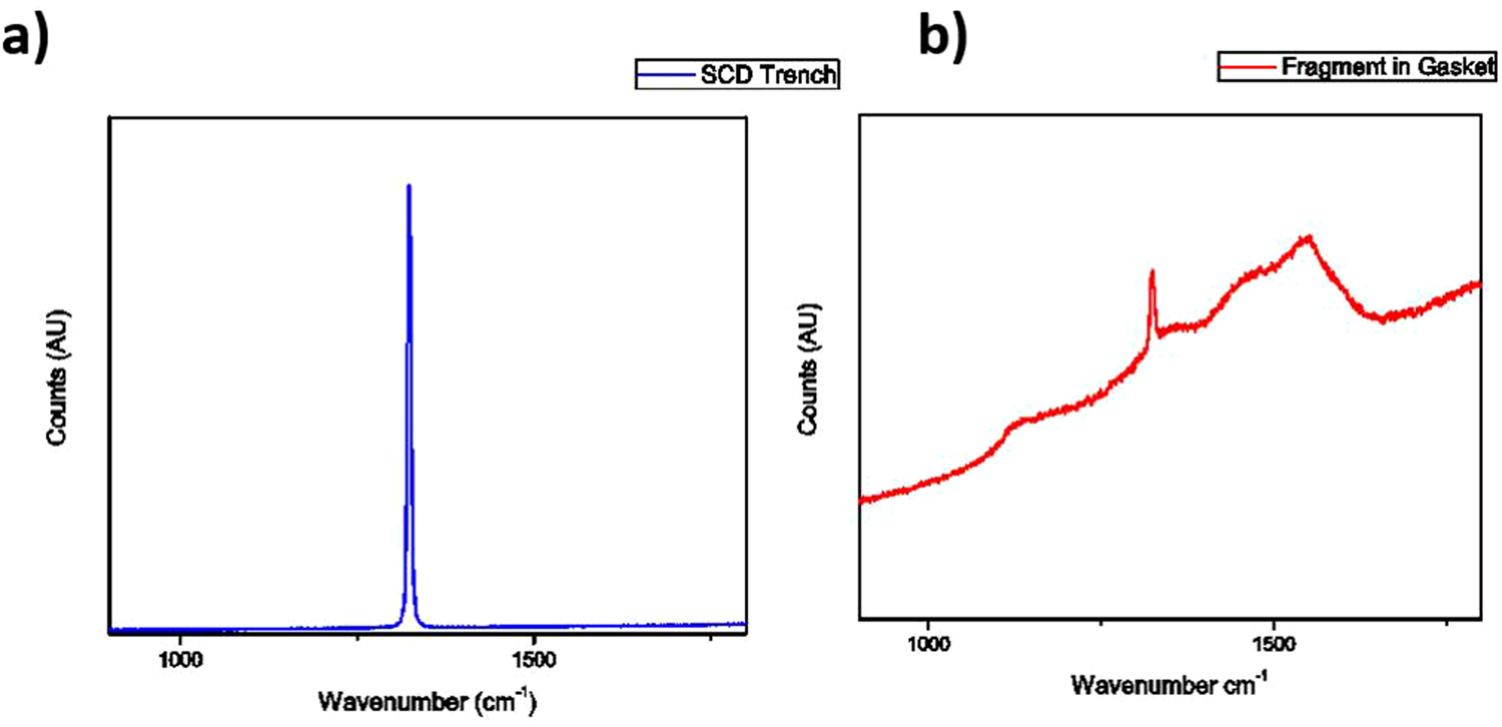
Raman spectra of: (**a**) the center of the trench in the single crystal diamond anvil. The well-defined narrow peak at 1332 cm^−1^ indicates high quality SCD, with no visible signal from sp^2^ bonded carbon present in NCD. (**b**) The NCD diamond fragment after pressure treatment embedded in the steel gasket showing a Raman signal characteristic of NCD along with 1332 cm^−1^ peak from sp^3^ bonded carbon from SCD.

Further confirmation that failure occurred in the bulk of the SCD is provided by Raman scans of the diamond fragment embedded into the gasket (Fig. 7). The Raman signal of the fragment shows a SCD peak at 1332 cm^−1^, in combination with a characteristic NCD spectrum. The Raman spectrum of the NCD fragment in Fig. 7b is identical to the Raman spectrum of as-grown NCD in Fig. 2 confirming that no change occurred in the structure of NCD during the pressure treatment. This is evidence that the detached diamond fragment consists of both NCD and SCD, which could only be possible if the anvil experienced failure in the bulk of the single crystal. Additionally, Raman scans of the trench in the SCD anvil showed no remnant NCD signal, indicating that failure occurred completely in the bulk of the SCD (Fig. 7). The maximum pressure point is located in the NCD micro-anvil (Fig. 4), however, failure in the diamond anvil is at the highest shear stress region that is generated in the interior of the anvil due to variation of axial and radial stresses as a function of depth below the NCD surface. Therefore, a likely cause of failure in SCD is that the location of maximum shear stress during high pressure experiments is located beneath the center of the culet surface in the bulk of the SCD, as predicted in literature through finite element modeling^20^.

## Conclusions

Spatially localized nanocrystalline diamond has been grown on single crystal diamond substrates are characterized by Raman spectroscopy, Scanning Electron Microscopy, and Atomic Force Microscopy and utilized in studies on materials under extreme conditions. Rapid nucleation of nanocrystalline diamond on single crystal diamond anvils has been demonstrated without implementing any pre-growth surface seeding. Grown NCD films tend to coalesce into spherical/hemispherical like structures, and appear to exhibit a geometrical limit in the potential of the total dimensions of growth that can be achieved. This is likely dependent on the initial geometry of the designated growth region.

The results are highly encouraging in supporting the continued use of NCD-SCD combinations in high pressure experiments. Not only have our experiments demonstrated substantial enhancement in the pressure generating capabilities of traditional SCD anvils to 0.5 TPa range, interfacial adhesive strength of NCD-SCD appears to be substantial and on the order of the cohesive bond strength in bulk single crystal. The blank experiment carried out with identical flat diamonds without the NCD stage only generated 75 GPa pressure. The examination of NCD micro-anvils that show detachment during compression/decompression in diamond anvil cell devices demonstrate that the failure occurs in the SCD substrate and the NCD/SCD interface can survive ultrahigh shear stresses. Further studies on the manipulation of grain size and adhesive strength of NCD-SCD will be highly useful in the optimization of grown NCD micro-anvils for applications in high-pressure research.
